# Novel taxa and species diversity of *Cordyceps* sensu lato (Hypocreales, Ascomycota) developing on wireworms (Elateroidea and Tenebrionoidea, Coleoptera)

**DOI:** 10.3897/mycokeys.78.61836

**Published:** 2021-03-29

**Authors:** Ling-Sheng Zha, Vadim Yu Kryukov, Jian-Hua Ding, Rajesh Jeewon, Putarak Chomnunti

**Affiliations:** 1 School of Life Sciences, Huaibei Normal University, Huaibei 235000, P.R. China; 2 School of Sciences, Mae Fah Luang University, Chiang Rai 57100, Thailand; 3 Center of Excellence in Fungal Research, Mae Fah Luang University, Chiang Rai 57100, Thailand; 4 Institute of Systematics and Ecology of Animals, Siberian Branch of Russian Academy of Sciences, Frunze str., 11, Novosibirsk 630091, Russia; 5 Department of Health Sciences, Faculty of Medicine and Health Sciences, University of Mauritius, Reduit 80837, Mauritius

**Keywords:** Two new species, Elateridae, molecular phylogeny, *
Ophiocordyceps
*, taxonomy, Tenebrionidae

## Abstract

Species of *Cordyceps* sensu lato (Hypocreales, Sordariomycetes) have always attracted much scientific attention for their abundant species diversity, important medicinal values and biological control applications. The insect superfamilies Elateroidea and Tenebrionoidea are two large groups of Coleoptera and their larvae are generally called wireworms. Most wireworms inhabit humid soil or fallen wood and are often infected with *Cordyceps* s.l. However, the species diversity of *Cordyceps* s.l. on Elateroidea and Tenebrionoidea is poorly known. In the present work, we summarise taxonomic information of 63 *Cordyceps* s.l. species that have been reported as pathogens of wireworms. We review their hosts and geographic distributions and provide taxonomic notes for species. Of those, 60 fungal species are accepted as natural pathogens of wireworms and three species (*Cordyceps militaris*, *Ophiocordyceps ferruginosa* and *O. variabilis*) are excluded. Two new species, *O. borealis* from Russia (Primorsky Krai) and *O. spicatus* from China (Guizhou), are described and compared with their closest allies. *Polycephalomyces formosus* is also described because it is reported as a pathogen of wireworms for the first time. Phylogeny was reconstructed from a combined dataset, comprising SSU, LSU and TEF1-α gene sequences. The results, presented in this study, support the establishment of the new species and confirm the identification of *P. formosus*.

## Introduction

The superfamilies Elateroidea and Tenebrionoidea are two large groups of Coleoptera. Species within these superfamilies are phytophagous, xylophagous, saprophagous or omnivorous and most of them are important agricultural pests (Gullan and Cranston 2010; Ren et al. 2016). Elateroidea larvae are the well-known wireworms, closely resembling Tenebrionoidea larvae which are known as mealworms or pseudo-wireworms (Ren et al. 2016). As a result, in practice, larvae of both Elateroidea and Tenebrionoidea are generally referred to as wireworms. Most wireworms inhabit humid soil, humus layer or decayed wood and are, thus, easily encountered and infected with entomopathogenic fungi (Kabaluk et al. 2017; Rogge et al. 2017).

*Cordyceps* sensu lato (Hypocreales, Sordariomycetes) is a well-known group of entomopathogenic fungi. Previously, most species of this group were assigned to the previous *Cordyceps* Fr. genus, so they had commonly been called ‘*Cordyceps*’. It was not until 2007 that Sung et al. revised the classification system of this group, based on substantial molecular and morphological data. In the new classification system, all these fungi are assigned to three families (Cordycipitaceae, Ophiocordycipitaceae and, in part, Clavicipitaceae) and only a few species were retained in the revised *Cordyceps* Fr. emend. G.H. Sung et al. genus (Sung et al. 2007). As a result, the concept of ‘*Cordyceps*’ has been extended from the previous genus *Cordyceps* Fr. to *Cordyceps* s.l. So far, more than 1000 *Cordyceps* s.l. species have been reported (Wei et al. 2020) and these entomopathogenic hypocrealean fungi are widely distributed in all terrestrial regions (except Antarctica), especially tropics and subtropics (Kobayasi 1941; Sung et al. 2007).

*Ophiocordyceps* Petch and *Polycephalomyces* Kobayasi are two morphologically, phylogenetically and ecologically closely-related genera placed in Ophiocordycipitaceae. They produce rigid, pliant or wiry stipes that are usually darkly coloured; their asexual morphs are mainly *Hirsutella*-like, but phialides of *Polycephalomyces* lack the swollen base and are concentrated at the tips of synnemata; and they are typically found on hosts buried in soil or in rotting wood, especially wireworms (Sung et al. 2007; Kepler et al. 2013). *Ophiocordyceps* is the largest genus of *Cordyceps* s.l., with *O. blattae* (Petch) Petch as the type species, linking with *Didymobotryopsis*-, *Hirsutella*-, *Hymenostilbe*-, *Sorosporella*-, *Synnematium*- and *Troglobiomyces*-like asexual states (Quandt et al. 2014) and currently comprising approximately 200 species (Wei et al. 2020). *Polycephalomyces*, with *P. formosus* Kobayasi as its type and linking with *Acremonium*-, *Hirsutella*- and *Polycephalomyces*-like asexual states, includes 19 known species thus far, some of which are found on stromata of *Ophiocordyceps* spp. (Kepler et al. 2013; Wang 2016; Index Fungorum 2021).

In nature, *Cordyceps* s.l. species develop mainly on insects, spiders, other *Cordyceps* s.l. species and hypogeous fungi of the genus *Elaphomyces*. These ascomycetes can reproduce via ascospores, conidia and mycelia that generally inhabit soil, plants, invertebrates, nematodes, mushrooms and other organisms (Zha et al. 2020). The ecology and habits of different host groups are generally different and this often determines the species specificity of *Cordyceps* s.l. on them. As a result, in practice, *Cordyceps* s.l. species have commonly been classified according to their host groups. With respect to the taxonomy of *Cordyceps* s.l. on insects, early systematic work mainly came from Petch (e.g. 1934), Kobayasi (e.g. 1941) and Shimizu (1997) who all classified *Cordyceps* s.l. species according to their host orders. Later, Shrestha et al. (2016, 2017) reviewed *Cordyceps* s.l. species on their Coleoptera, Lepidoptera, Hymenoptera and Hemiptera hosts. Recently, Zha et al. (2020) systematically studied the Orthoptera hosts and investigated the relationships with their pathogens.

A diverse range of *Cordyceps* s.l. species have been reported as pathogens of wireworms. Due to the difficulities in identifying wireworms, hosts of these fungal species have generally been recorded as Elateridae larvae, Tenebrionidae larvae or Coleoptera larvae (e.g. Petch 1933, 1937; Kobayasi 1941; Kobayasi and Shimizu 1982b, 1983). Shimizu (1997) provided beautiful drawings for many *Cordyceps* s.l. species, which included more than 30 species on wireworms and wireworm-like insects. A recent report for wireworm-infecting *Cordyceps* s.l. involved only 20 species (Shrestha et al. 2016), which is fewer than the number recorded by Shimizu (1997). It should be noticed that these fungi affect the populations of wireworms and have the potential to control these agricultural pests (Barsics et al. 2013; Rogge et al. 2017). Therefore, we need a deeper knowledge of species diversity, taxonomy, distribution and lifestyle of these wireworm-infecting *Cordyceps* s.l.

In this study, the species diversity of wireworm-infecting *Cordyceps* s.l. (Elateroidea and Tenebrionoidea) is reviewed. We discuss their hosts and geographic distribution and provide taxonomic notes for species. In addition, we describe two new members of this group, *Ophiocordyceps borealis* sp. nov. and *O. spicatus* sp. nov. *Polycephalomyces formosus* Kobayasi is also described because it represents the first report of this species on wireworms (Elateroidea). We reconstructed a multilocus (SSU, LSU and TEF1-α) phylogeny to support morphological results.

## Material and methods

### Sample collections and morphological studies

Wireworm-infecting species of *Cordyceps* s.l. were collected from south-western China and the Russian Far East. Specimens were placed in plastic boxes and carried to the laboratory for further study. The macro-characteristics and ecology were photographed using a Nikon Coolpix P520 camera in the field. Specimens were examined and photographed using an Optec SZ660 stereo dissecting microscope and a Nikon Eclipse 80i compound microscope connected with a Canon EOS 600D camera. Microscopic measurements were made using Tarosoft (R) Image Framework software. Images were processed using Adobe Photoshop CS v. 8.0.1 (Adobe Systems Incorporated, San Jose, California, USA). Voucher specimens are deposited in the Fungarium of the Centre of Excellence in Fungal Research, Mae Fah Luang University (MFLU), Chiang Rai, Thailand and the Herbarium of Guizhou University (GACP), Guiyang, China.

### DNA extraction, sequencing, sequence assembly and alignment

Total DNA was extracted from dried specimens using E.Z.N.A.TM Fungal DNA MiniKit (Omega Biotech, CA, USA). The ribosomal internal transcribed spacers (ITS), small and large subunits (SSU and LSU) and translation elongation factor 1α (TEF1-α) genes were amplified and sequenced using the PCR programmes and primer pairs listed in Table 1. PCR amplification reactions were performed in an ABI 2720 thermal cycler (Applied Biosystems, Foster City, CA, USA). PCR products were purified using Bioteke’s Purification Kit (Bioteke Corporation, Beijing, China) and were sequenced using an ABI 3730 DNA analyser and an ABI BigDye 3.1 terminator cycle sequencing kit (Sangon Co., Shanghai, China). Sequences were aligned and assembled visually and manually using Clustalx1.81, Chromas230, ContigExpress and MEGA6 software.

**Table 1.** Primers and PCR programmes used in this study (White et al. 1990, Spatafora et al. 2006, Ban et al. 2015).

Locus	Primers	PCR programs (optimised)
ITS	ITS4: 5’-TCCTCCGCTTATTGATATGC-3’	(94 °C for 30 s, 51 °C for 50 s, 72 °C for 45 s) × 33 cycles
	ITS5: 5’-GGAAGTAAAAGTCGTAACAAGG-3’	
SSU	NS1: 5’-GTAGTCATATGCTTGTCTC-3’	(94 °C for 30 s, 51 °C for 30 s, 72 °C for 2 min) × 33 cycles
	NS4: 5’-CTTCCGTCAATTCCTTTAAG-3’	
LSU	LROR: 5’-ACCCGCTGAACTTAAGC-3’	(94 °C for 30 s, 55 °C for 30 s, 72 °C for 1 min) × 30 cycles
	LR5: 5’-TCCTGAGGGAAACTTCG-3’	
TEF1-α	EF1-983F: 5’-GCYCCYGGHCAYCGTGAYTTYAT-3’	(94 °C for 1 min, 55 °C for 30 s, 72 °C for 2 min) × 35 cycles
	EF1-2218R: 5’-ATGACACCRACRGCRACRGTYTG-3’	

### Construction of molecular phylogenetic trees

BLAST searches were performed to reveal the closest matches in the GenBank database that would allow the selection of appropriate taxa for phylogenetic analyses. Each gene region was independently aligned and improved manually, then the SSU, LSU and TEF1-α gene sequences were combined to form a concatenated dataset. The ITS region was not included in our multilocus analyses because of: 1) insufficient ITS sequence data (Table 2) which may lead to inaccurate phylogenetic results; 2) distinct different rate of evolution from SSU, LSU and TEF genes and with many irregular insertions and deletions of bases. Maximum Likelihood (ML), Maximum Parsimony (MP) and Bayesian Inference (BI) analyses were performed using the concatenated sequence dataset. Sequence information of the three described species and their allies is listed in Table 2.

**Table 2.** Sequence information of samples used in this study. Our sequencing results are displayed in bold.

Fungal species	Specimen/ strain No.	Host/substratum	ITS	SSU	LUS	TEF1–α	References
*Cordyceps militaris* (outgroup)	OSC 93623	Lepidoptera (larva)	JN049825	AY184977	AY184966	DQ522332	Kepler et al. (2012)
*Ophiocordyceps annulata*	CEM303	Coleoptera	–	KJ878915	KJ878881	KJ878962	Quandt et al. (2014)
*O. aphodii*	ARSEF 5498	Coleoptera	–	DQ522541	DQ518755	DQ522323	Spatafora et al. (2007)
***O. borealis* sp. nov.**	**MFLU 18-0163**	**Coleoptera: Elateroidea (larva)**	**MK863251**	**MK863044**	**MK863051**	**MK860189**	**This study**
	**GACP R16002**	**Coleoptera: Elateroidea (larva)**	**MK863252**	**MK863045**	**MK863052**	**MK860190**	
	**GACP R16003**	**Coleoptera: Elateroidea (larva)**	**MK863253**	**MK863046**	**MK863053**	**MK860191**	
*O. clavata*	NBRC 106962	Coleoptera (larva)	JN943328	JN941726	JN941415	AB968587	Schoch et al. (2012)
*O. cossidarum*	MFLU 17-0752	Lepidoptera (larva)	–	MF398186	MF398187	MF928403	Hyde et al. (2018)
*O. entomorrhiza*	KEW 53484	Lepidoptera	JN049850	EF468954	EF468809	EF468749	Quandt et al. (2014)
*O. formosana*	MFLU 15-3889	Tenebrionoidea (larva)	–	–	–	KU854950	Li et al. (2016)
*O. formosana*	MFLU 15-3888	Tenebrionoidea (larva)	–	KU854951	–	KU854949	Li et al. (2016)
*O. konnoana*	EFCC 7315	Coleoptera (larva)	–	EF468959	–	EF468753	Sung et al. (2007)
*O. lanpingensis*	YHOS0707	Lepidoptera: Hepialidae (larva)	–	KC417459	KC417461	KC417463	Chen et al. (2013)
*O. longissima*	NBRC 108989	Hemiptera (cicada nymph)	AB968407	AB968394	AB968421	AB968585	Sanjuan et al. (2015)
*O. macroacicularis*	NBRC 105888	Lepidoptera (larva)	AB968401	AB968389	AB968417	AB968575	Ban et al. (2015)
*O. melolonthae*	OSC 110993	Coleoptera: Scarabeidae (larva)	–	DQ522548	DQ518762	DQ522331	Spatafora et al. (2007)
*O. nigra*	TNS 16252	Hemiptera	–	KJ878941	KJ878906	KJ878986	Quandt et al. (2014)
*O. nigrella*	EFCC 9247	Lepidoptera (larva)	JN049853	EF468963	EF468818	EF468758	Sung et al. (2007)
*O. purpureostromata*	TNS F18430	Coleoptera	–	KJ878931	KJ878897	KJ878977	Quandt et al. (2014)
*O. ravenelii*	OSC 110995	Coleoptera (larva)	–	DQ522550	DQ518764	DQ522334	Spatafora et al. (2007)
*O. robertsii*	KEW 27083	Lepidoptera: Hepialidae (larva)	AJ309335	–	EF468826	EF468766	Sung et al. (2007)
*O. sinensis*	EFCC 7287	Lepidoptera (pupa)	JN049854	EF468971	EF468827	EF468767	Sung et al. (2007)
*O. sobolifera*	NBRC 106967	Hemiptera (cicada nymph)	AB968409	AB968395	AB968422	AB968590	Ban et al. (2015)
***O. spicatus* sp. nov.**	**MFLU 18-0164**	**Coleoptera: Tenebrionoidea (larva)**	**MK863254**	**MK863047**	**MK863054**	**MK860192**	**This study**
*O. variabilis*	OSC 111003	Diptera (larva)	–	EF468985	EF468839.	EF468779	Sung et al. (2007)
*O. xuefengensis*	GZUH2012HN19	Lepidoptera: *Endoclita nodus* (larva)	KC631803	KC631788	–	KC631794	Wen et al. (2013)
*Paraisaria amazonica*	Ophama2026	Orthoptera: Acrididae (nymph)	–	KJ917562	KJ917571	KM411989	Sanjuan et al. (2015)
*P. coenomyiae*	NBRC 108993	Diptera: *Coenomyia* (larva)	AB968396	AB968384	AB968412	AB968570	Ban et al. (2015)
*P. gracilis*	EFCC 8572	Lepidoptera (larva)	JN049851	EF468956	EF468811	EF468751	Kepler et al. (2012)
*P. heteropoda*	OSC106404	Hemiptera (cicada nymph)	–	AY489690	AY489722	AY489617	Castlebury et al. (2004)
***Polycephalomyces formosus***	**MFLU 18-0162**	***Ophiocordyceps* sp. (stroma) on an Elateroidea larva**	**MK863250**	**MK863043**	**MK863050**	**MK860188**	**This study**
*P. formosus*	ARSEF 1424	Coleoptera	KF049661	KF049615	KF049634	DQ118754	Chaverri et al. (2005)
*P. lianzhouensis*	GIMYY9603	Lepidoptera	EU149922	KF226249	KF226250	KF226252	Wang et al. (2014)
*P. ramosopulvinatus*	EFCC 5566	Hemiptera	KF049658	–	KF049627	KF049682	Kepler et al. (2013)
*P. sinensis*	CN 80-2	*O. sinensis* (stroma)	HQ832884	HQ832887	HQ832886	HQ832890	Wang et al. (2012)
*P. tomentosus*	BL 4	Trichiales	KF049666	KF049623	KF049641	KF049697	Kepler et al. (2013)
*P. yunnanensis*	YHHPY1006	*O. nutans* (stroma)	KF977849	–	–	KF977851	Wang et al. (2015)

Maximum Likelihood (ML) analysis was done via the CIPRES Science Gateway platform (Miller et al. 2010) using RAxML-HPC2 on XSEDE (8.2.10) with the GTRGAMMA nucleotide substitution model and 1000 bootstrap iterations (Jeewon et al. 2003; Hongsanan et al. 2017). An MP tree was constructed with PAUP* 4.0b10 (Swofford 2002) using the heuristic search option with TBR branch swapping and bootstrapping with 1,000 replicates (Cai et al. 2006; Tang et al. 2007). BI analysis was conducted using MrBayes v. 3.1.2 with Markov Chain Monte Carlo sampling to calculate posterior probabilities (PP) (four simultaneous Markov chains running for 1,000,000 generations; sampling every 100 generations, first 25% of sampled trees discarded) (Rannala and Yang 1996).

## Results

### Molecular phylogeny of the three described species

**Figure 1.**
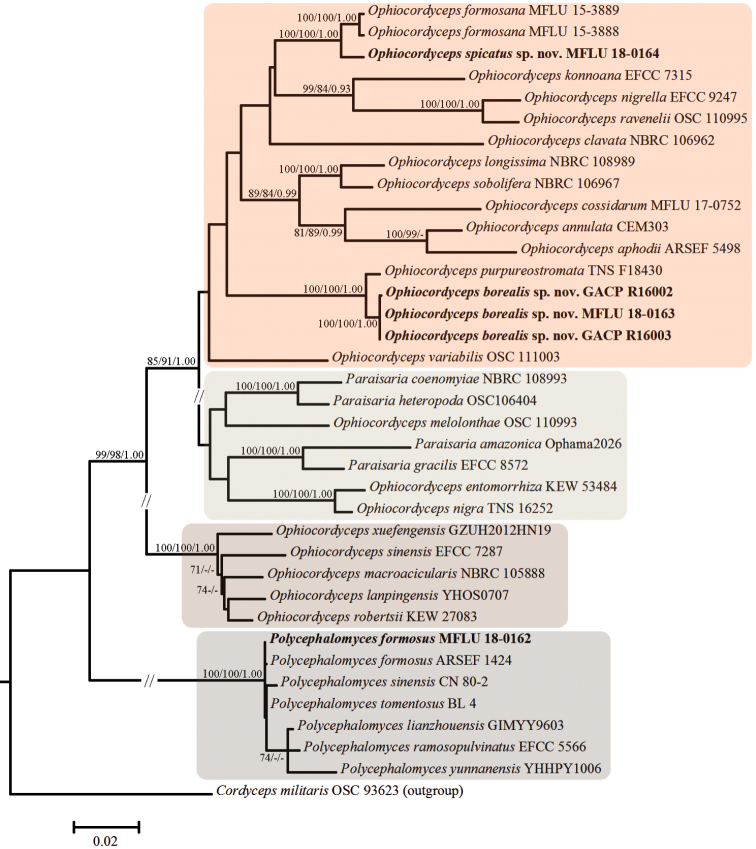
Maximum Likelihood (ML) tree of *Ophiocordyceps borealis* sp. nov., *O. spicatus* sp. nov. and their allies inferred from a combined SSU, LSU and TEF1-α gene dataset. Bootstrap support values of ML and Maximum Parsimony (MP) > 60% and posterior probabilities (PP) of Bayesian Inference > 0.9, are indicated above the nodes and separated by ‘/’ (ML/MP/PP).

The combined concatenated dataset included 36 samples including 32 species of Ophiocordycipitaceae (*Ophiocordyceps*, *Paraisaria* and *Polycephalomyces*) as ingroups and *Cordyceps militaris* (L.) Fr. (strain OSC 93623, Kepler et al. 2012) as the outgroup. The aligned dataset was deposited in the TreeBASE database (http://purl.org/phylo/treebase/phylows/study/TB2:S26977?x-access-code=cb3474ce0fd0327526b6fd2465d6c53d&format=html). The aligned dataset was composed of 2,843/2,837 (including/excluding outgroup) characters (including gaps), of which 740/681 were variable and 527/520 were parsimony-informative. ML, MP and BI analyses resulted in phylogenies with similar topologies and the best-scoring ML tree (–lnL= 15804.4393) is shown in Fig. 1.

According to the phylogenetic tree (Fig. 1), three *Ophiocordyceps borealis* sp. nov. samples (specimens MFLU 18-0163, GACP R16002 and GACP R1600) group together (100% ML/100% MP/1.00 PP) and are related to, but phylogenetically distinct from, *O. purpureostromata* (specimen TNS F18430). *Ophiocordyceps spicatus* sp. nov. (specimen MFLU 18-0164) constitutes a strongly supported independent lineage and is related to *O. formosana*. The two *Polycephalomyces formosus* samples (specimens MFLU 18-0162 and ARSEF 1424) group together and are related to *P. sinensis* (specimen CN 80-2) and *P. tomentosus* (specimen BL 4).

### New species and new record of *Cordyceps* s.l. developing on wireworms

#### Ophiocordyceps borealis

Taxon classificationFungiHypocrealesOphiocordycipitaceae

L.S. Zha & P. Chomnunti sp. nov.

38B3409D-DD5D-54BF-8B40-6D26A0358542

Fig. 2

##### Etymology.

Referring to the region (south of boreal zone of the Russian Far East) from where the species was collected.

##### Sexual morph.

Parasitising Elateroidea larvae (Coleoptera) living in fallen wood. The larvae are cylindrical, 11 mm long and 1.1–1.3 mm thick, yellowish-brown; their body cavity stuffed with milky yellow mycelia and their intersegmental membranes covered with many milky yellow and flocculent funiculi. *Stromata* arising from any part of larval body, single or paired, unbranched. Stipe grey, slender and cylindrical, fibrous and flexible, curved more or less, 10–13 mm long and 0.25–0.6 mm thick, surface relatively smooth but with many longitudinal wrinkles, apex pointed. *Fertile part* irregularly attached on one side of the surface of distal part of stipe, which resembles a mass of insect eggs that are clustered together or separated into several lumps; substrate layer milky white, surface milky yellow accompanied by lavender and dotted with numerous black ostioles. *Perithecia* immersed, densely arranged, obliquely or at right angles to the surface of stipe, pyriform, neck unconspicuous, 220–290 × 120–150 µm and their tops obtuse; walls dark brown and 25–32 µm thick; ostioles slightly thickened and slightly protruding over the surface of fertile part. *Asci* cylindrical, 6–8 µm in diameter; caps hemispherical, 5–6 (*x*– = 5.5, n = 30) µm wide and 3.5–5 (*x*– = 4.2, n = 30) µm high. *Ascospores* filiform and elongate, multi-septate (far more than 3), not easy to break into part-spores; part-spores cylindrical, truncated at both ends, 10–15 (*x*– = 12.2, n = 30) × 2 μm. **Asexual morph.** Unknown.

**Figure 2.**
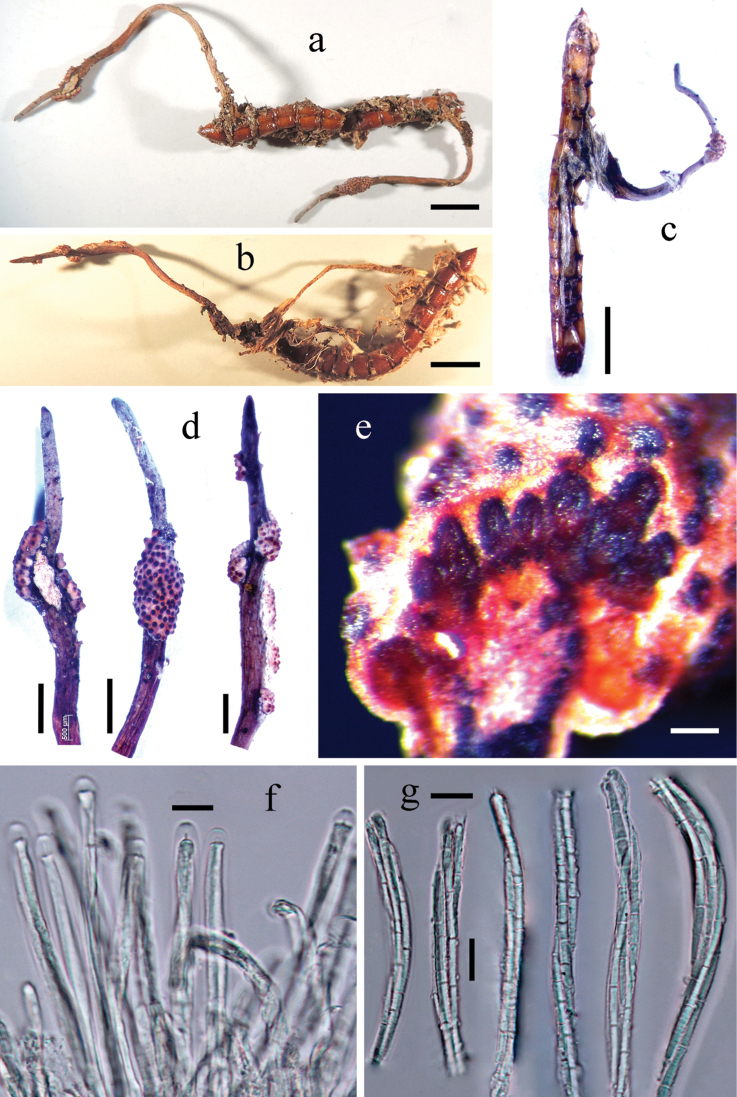
*Ophiocordyceps borealis***a–c** stromata arising from the different parts of larval bodies **d** apical ends of stromata **e** transverse section of fertile part, on which densely arranged perithecia are shown **f** asci **g** ascospores. Scale bars: 2 mm (**a–c**); 1 mm (**d**); 100 µm (**e**), 10 µm (**f, g**).

##### Material examined.

Russia, the Russian Far East, Primorskiy Krai, National Park Land of the Leopard, Natural Reserve Kedrovaya Pad, 43°05'53.8"N, 131°33'17.8"E, 10 August 2016, Oksana Tomilova & Vadim Yu Kryukov (MFLU 18-0163, **holotype**; GACP R16002 and GACP R16003, **paratypes**).

##### Known distribution.

Russia (Primorskiy Krai).

##### Hosts.

Growing on Elateroidea larvae (Coleoptera) living in fallen wood in a deciduous forest.

##### Notes.

The new species is morphologically similar to *O. purpureostromata* (≡ *C. purpureostromata*), but their stipes and ascospores are distinct. In *O. purpureostromata*, stipe is thicker (0.6–1 mm in diameter) and has hairs (0.25–0.6 mm in diameter and without hair in *O. borealis*), ascospores are only 65–75 × 10 µm long and 3-septate (elongate and far more than 3-septate in *O. borealis*) and part-spores are 13–23 µm long (10–15 µm long in *O. borealis*) (Kobayasi and Shimizu 1980b).

Nucleotide sequences of *O. borealis* are most similar to those of *O. purpureostromata* (specimen TNS F18430, Quandt et al. 2014), but there is 2.3% bp difference across the 804 bp in TEF1-α, 0.5% bp difference across the 845 bp in LSU and 0.1% bp difference across 1,061 bp in SSU. ITS of *O. borealis* is > 14.1% different to all ITS available in GenBank (ITS are not available for *O. purpureostromata*). On the phylogenetic tree, the new species is also nearest (100% ML/100% MP/1.00 PP) to *O. purpureostromata*, but they form into two distinct branches which support them being two separate species (Fig. 1).

#### Ophiocordyceps spicatus

Taxon classificationFungiHypocrealesOphiocordycipitaceae

L.S. Zha & P. Chomnunti sp. nov.

F73D189B-B2DF-56B5-A9BE-0A32B96B3AE8

Fig. 3

##### Etymology.

Referring to the spicate fertile head.

##### Sexual morph.

Parasitising a Tenebrionoidea larva (Coleoptera) living in humid and decayed wood. The larva is cylindrical, 7.5 mm long and 1.0–1.1 mm thick, yellowish-brown. White mycelia stuff the body cavity, also partially cover the intersegmental membranes of the body surface. *Stroma* arising from the first quarter of the larval body, single, fleshy, 5 mm in length. Stipe yellow, cylindrical, 3.5 mm long and 0.35–0.4 mm thick, surface rough and pubescent. *Fertile head* spicate, unbranched, orange, 1.5 mm long and 0.5–0.7 mm thick, obviously differentiated from stipe; its surface rugged and consisting of many humps (outer portions of perithecia), tops of the humps obtuse and with opening ostioles, darker in colour. *Perithecia* partially immersed and obliquely or at right angles to the surface of stipe, broadly pyriform, 200–250 × 170–200 μm; walls 25–35 μm thick. *Asci* cylindrical, 5–9 μm thick, middle part wider than two terminal parts; caps hemispheric, 4.6–5.3 (*x*– = 4.9, n = 30) μm wide and 4.0–4.6 (*x*– = 4.3, n = 30) μm high. *Ascospores* filiform; part-spores cylindrical, truncated at both ends, 3.5–6.5 (*x*– = 4.7, n = 30) μm long and 1.7–2.0 μm thick. **Asexual morph.** Unknown.

**Figure 3.**
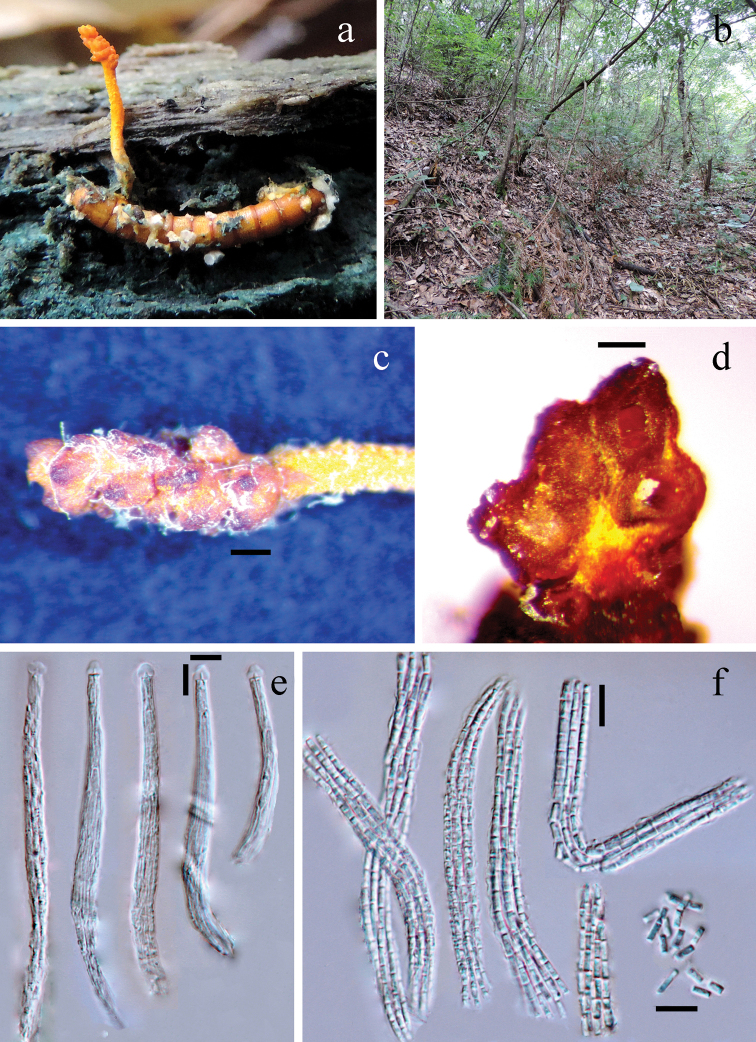
*Ophiocordyceps spicatus* (MFLU 18-0164) **a** infected larva in decayed wood **b** habitat environment **c** fertile head of stroma **d** transverse section of fertile head, on which sparse arranged perithecia are shown **e** Asci **f** Ascospores and part-spores. Scale bars: 200 µm (**c**); 100 µm (**d**) 10 µm (**e, f**).

##### Material examined.

China, Guizhou Province, Leishan County, Leigongshan Mountain, 26°22'18"N, 108°11'28"E, 1430 m alt., 2 August 2016, Ling-Sheng Zha (MFLU 18-0164, **holotype)**.

##### Known distribution.

China (Guizhou).

##### Host.

Growing on a Tenebrionoidea larva (Coleoptera) living in humid and decayed wood in a broad-leaved forest.

##### Notes.

*Ophiocordyceps spicatus* is morphologically somewhat similar to *O. formosana* (Kobayasi and Shimizu 1981; Li et al. 2016), but it has a much smaller stroma (stipes 6–10 (or 19–37) mm long and 1.5–1.7 (or 2–4) mm wide in *O. formosana*), a spicate and rugged fertile head (surface entire and flattened, never spicate or rugged in *O. formosana*) and partially immersed perithecia (immersed in *O. formosana*).

Nucleotide sequences of *O. spicatus* are most similar to those of *O. formosana*, but there is 5.2% bp difference in ITS, 2.0% bp difference in TEF1-α and 0.1% bp difference in SSU (LSU rDNA sequence unavailable for *O. formosana*). LSU of *O. spicatus* is > 5.6% bp different to all LSU available in GeneBank. Additionally, on the phylogenetic tree, *O. spicatus* is closely related (100% ML/100% MP/1.00 PP) to *O. formosana*, but they form into two distinct branches which also support them being two separate species (Fig. 1).

#### Polycephalomyces formosus

Taxon classificationFungiHypocreales

Kobayasi

6641EF85-1082-53C7-9A0B-F8C466B84E75

289806

Fig. 4

##### Remarks.

*Polycephalomyces formosus* was reported on Coleoptera larvae, stromata of *Ophiocordyceps barnesii* (Thwaites) G.H. Sung et al., *O. falcata* (Berk.) G.H. Sung et al. and *O. cantharelloides* (Samson & H.C. Evans) G.H. Sung et al. and distributed in Ecuador, Japan and Sri Lanka (Kobayasi 1941; Samson and Evans 1985; Wang 2016). We collected a *P. formosus*-like specimen on the stroma of *Ophiocordyceps* sp. on an Elateroidea larva from Guizhou, China. Morphological and phylogenetic data showed that it is *P. formosus*. This is the first report of *P. formosus* on wireworms.

##### Asexual morph.

Growing on the stroma of *Ophiocordyceps* sp. on an Elateroidea larva. Stroma single, arising from the body end of the host larva, unbranched. The larva reddish-brown, cylindrical, 21 × 1.3–1.6 mm, intersegmental membranes conspicuous. Stipe of the stroma shiny black, stiff, band-like, but twisted and deeply wrinkled (dry specimen), more than 20 mm long and 1.0–1.3 mm thick, surface smooth (the fertile head was missing). *Synnemata* solitary or caespitose, arising from the intersegmental membranes of the larva and the surface of the stroma, mostly unbranched, generally straight, capitate, 1–3.5 mm long and 50–600 µm thick. Stipe basally broad and compressed, then gradually cylindrical upwards, white, greyish-white to yellowish-brown, surface smooth. *Fertile head* (including spore mass) abruptly expanded, ellipsoidal, 100–300 × 80–250 µm, located at the top of every synnema and distinctly separated from the stipe. Spore mass covers the surface of every fertile head, 15–25 µm thick, yellowish-brown and composed of hymenia. *Phialides* of two types, A-phialides produced on fertile heads, B-phialides arising laterally along the entire stipe. A-phialides 3–5 in terminal whorl on basal conidiophores, cylindrical to narrowly conical, straight or curved, non-uniform, 10–20 (*x*– = 15.1, n = 30) µm long and 1.5–2 µm (*x*– = 1.7, n = 30) wide, basally and terminally narrow, neck narrow to 0.5 µm, collarettes and periclinal thickening not visible; *A-conidia* obovate to obpyriform, smooth-walled, hyaline, 2.1–3.2 (*x*– = 2.6, n = 30) µm long and 1.5–2.2 (*x*– = 1.8, n = 30) µm wide. B-phialides single or in terminal whorls of 2–3 on basal conidiophores, straight, symmetrical or asymmetrical, hyaline, generally cylindrical, 10–25 (*x*– = 17, n = 30) µm long, 2–3.5 (*x*– = 2.8, n = 30) µm thick at the base, 0.5–0.8 (*x*– = 0.65, n =30) µm thick at the end, collarettes and periclinal thickening not visible; *B-conidia* fusiform, hyaline, smooth-walled, 3.2–6.0 (*x*– = 4.6, n = 30) µm long and 1–1.8 (*x*– = 1.4, n = 30) µm wide. **Sexual morph.** Not observed.

**Figure 4.**
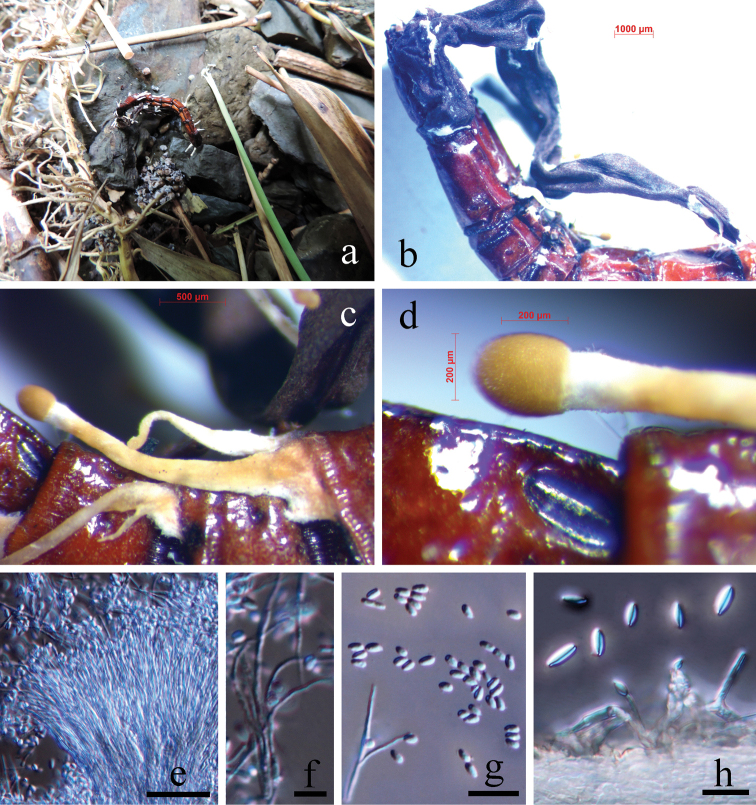
*Polycephalomyces formosus* (MFLU 18-0162) **a** collected on the ground in a bamboo forest **b** produced on the stroma of *Ophiocordyceps* sp. (the fertile head was missing) on an Elateroidea larva **c, d** synnemata **e–g** A-type phialides and A-type conidia **h** B-type phialides and B-type conidia. Scale bars: 20 µm (**e**); 5 µm (**f**); 10 µm (**g, h**).

##### Material examined.

CHINA, Guizhou, Tongzi County, Baiqing Natural Reserve, 28°52'31"N, 107°9'10"E, about 1300 m alt., 13 July 2016, Ling-Sheng Zha (MFLU 18-0162).

##### Notes.

*Polycephalomyces formosus* was originally described from Japan as: growing on Coleoptera larvae; synnemata solitary or caespitose, 1–3.5 mm long and 100–250 µm thick; spore mass covering the surface of the fertile head, 15–25 µm thick; A-phialides 3–4 in terminal whorl on basal conidiophores, cylindrical to narrowly conical, 10–20 × 1.5–2 µm, neck 0.5 µm; A-conidia obovate to obpyriform, 2.0–2.8 × 1.6–2.0 µm; B-conidia fusiform, 3.2–4.8 × 0.8–1.6 µm (Kobayasi 1941; Wang 2016). These characteristics are all consistent with our specimen. Sequences of SSU, ITS, LSU and TEF1-α are all identical to those of *P. formosus* (specimen ARSEF 1424); and in our phylogenetic tree, these two samples grouped together and have a same branch length (Fig. 1).

##### Host and ecology.

On the stroma of *Ophiocordyceps* sp. on an Elateroidea larva on the ground in a humid bamboo (*Chimonobambusa quadrangularis* (Franceschi) Makino) forest in Guizhou karst regions.

The larva might live in soil or decayed wood at first, but was then infected by *Ophiocordyceps* sp. and produced a sexual stroma. Following heavy rainfall, the host, together with the stroma of *Ophiocordyceps* sp., was washed away and exposed on the ground and at last, was parasitised by *Polycephalomyces formosus*. The fertile head of the stroma might have been lost during the floods.

### Annotated list of recorded *Cordyceps* s.l. species developing on wireworms

**Order Hypocreales Lindau**

**Family Cordycipitaceae Kreisel ex G.H. Sung, J.M. Sung, Hywel-Jones & Spatafora**

#### Akanthomyces lecanii

Taxon classificationFungiHypocrealesCordycipitaceae

(Zimm.) Spatafora, Kepler & B. Shrestha

B9753C35-1890-5013-8F4A-13A3988239BF

≡ Cephalosporium lecanii Zimm.
≡ Verticillium lecanii (Zimm.) Viégas
≡ Lecanicillium lecanii (Zimm.) Zare & W. Gams
= Cephalosporium lecanii f. coccorum (Petch) Bałazy
= Sporotrichum lichenicola Berk. & Broome
= Hirsutella confragosa Mains
= Torrubiella confragosa Mains
= Cordyceps confragosa (Mains) G.H. Sung, J.M. Sung, Hywel-Jones & Spatafora
= Cephalosporium coccorum Petch
= Verticillium coccorum (Petch) Westerd.
= Cephalosporium coccorum var. uredinis U.P. Singh & Pavgi
= Cephalosporium subclavatum Petch
For further doubtful synonyms, see Zare and Gams (2001).

##### Hosts.

Spiders, insects from various orders, including Coleoptera (e.g. Tenebrionidae: *Alphitobius diaperinus*); inhabiting phytopathogenic fungi and plant-parasitic nematodes (Humber and Hansen 2005; Shinya et al. 2008).

##### Distribution.

Widely distributed in tropical and temperate regions, for example: Dominican Republic, Jamaica, Indonesia, Peru, Sri Lanka, the West Indies, Turkey and USA (Zare and Gams 2001).

##### Notes.

The species was originally and frequently reported on scale insects (Hemiptera: Coccidae (syn. Lecaniidae)) (Zare and Gams 2001). Humber and Hansen (2005) listed its hosts involving spiders, many insect orders and found on the mushroom *Puccinia striiformis* (Pucciniaceae). The species was also found on phytopathogenic fungi and plant-parasitic nematodes (Shinya et al. 2008). Zare and Gams (2001) systematically studied the species and listed its synonyms. Kepler et al. (2017) rejected *Torrubiella* and *Lecanicillium* and transferred the species to *Akanthomyces*.

#### Beauveria bassiana

Taxon classificationFungiHypocrealesCordycipitaceae

sensu lato

878E533F-731E-5EA8-BD35-769F5D8D8ABE

##### Hosts.

Many insect orders, including Coleoptera (e.g. Elateroidea and Tenebrionoidea spp., Humber and Hansen 2005; Reddy et al. 2014; Sufyan et al. 2017); inhabiting soil, plant surfaces and plant internal tissues (Bamisile et al. 2018).

##### Distribution.

Widely distributed.

##### Note.

*Beauveria bassiana* sensu lato includes a large complex of cryptic species with wide host ranges, including many Coleoptera families (Rehner et al. 2011; Imoulan et al. 2017).

#### Cordyceps aurantiaca

Taxon classificationFungiHypocrealesCordycipitaceae

Lohwag

C4331352-35ED-55BB-97D6-6F37C5A36CA1

##### Hosts.

Elateridae larvae (Keissler and Lohwag 1937).

##### Known distribution.

China (Keissler and Lohwag 1937).

##### Note.

Taxonomically uncertain species which was described from the previous *Cordyceps* Fr. (differs from the current *Cordyceps* Fr. emend. G.H. Sung et al., same as below).

#### Cordyceps chiangdaoensis

Taxon classificationFungiHypocrealesCordycipitaceae

Tasanathai, Thanakitpipattana, Khonsanit & Luangsa-ard

15F26C9F-A6DE-592C-870C-F8811FECA1A6

##### Hosts.

Elateroidea or Tenebrionoidea larvae.

##### Known distribution.

Thailand (Tasanathai et al. 2016).

##### Note.

Hosts of the species were recorded as Coleoptera larvae (Tasanathai et al. 2016). According to the picture provided, the hosts are wireworms.

#### Cordyceps chishuiensis

Taxon classificationFungiHypocrealesCordycipitaceae

Z.Q. Liang & A.Y. Liu

1EC324BE-2B4A-5A72-9121-B2698036B8FD

##### Host.

Elateroidea or Tenebrionoidea larva.

##### Known distribution.

China (Guizhou) (Liang 2007).

##### Notes.

Taxonomically uncertain species from the previous *Cordyceps*. The species was originally reported on a wireworm (Liang 2007).

#### Cordyceps farinosa

Taxon classificationFungiHypocrealesCordycipitaceae

(Holmsk.) Kepler, B. Shrestha & Spatafora

364247D5-C695-5011-BB8A-0180BE374DAF

≡ Ramaria farinosa Holmsk.
≡ Clavaria farinosa (Holmsk.) Dicks.
≡ Corynoides farinosa (Holmsk.) Gray
≡ Isaria farinosa (Holmsk.) Fr.
≡ Spicaria farinosa (Holmsk.) Vuill.
≡ Penicillium farinosum (Holmsk.) Biourge
≡ Paecilomyces farinosus (Holmsk.) A.H.S. Br. & G. Sm.
For further doubtful synonyms, see Index Fungorum (2021).

##### Hosts.

Mites, spiders, insects from various orders, including Coleoptera (e.g. Tenebrionidae spp.); inhabiting soil, humus, plants, fungi and other organisms (Humber and Hansen 2005; Zimmermann 2008).

##### Distribution.

Widely distributed (Zimmermann 2008).

##### Note.

According to Domsch et al. (1980) and Zimmermann (2008), the species is ubiquitous in temperate and tropical zones.

#### Cordyceps fumosorosea

Taxon classificationFungiHypocrealesCordycipitaceae

(Wize) Kepler, B. Shrestha & Spatafora

2FF223AE-0773-5E9B-A2AF-89C5C04DEC3C

≡ Isaria fumosorosea Wize
≡ Spicaria fumosorosea (Wize) Vassiljevsky
≡ Paecilomyces fumosoroseus (Wize) A.H.S. Br. & G. Sm.
= Paecilomyces fumosoroseus var. beijingensis Q.X. Fang & Q.T. Chen

##### Hosts.

Mites, insects from various orders (e.g. Lagriidae and Tenebrionidae spp. in Tenebrionoidea) (Humber and Hansen 2005; Zimmermann 2008).

##### Distribution.

Widely distributed (Zimmermann 2008).

##### Note.

The species was previously confused with *C. farinosa* or regarded as a complex species (Zimmermann 2008).

#### Cordyceps huntii

Taxon classificationFungiHypocrealesCordycipitaceae

Giard [as ‘hunti’, ‘lunti’]

CFC1AA02-B58D-51B3-9878-3562FD941371

##### Host.

Elateridae larva (Massee 1899).

##### Known distribution.

Gaul (Massee 1899).

##### Notes.

Taxonomically uncertain species from the previous *Cordyceps*. Sung et al. (2007) treated it as a synonym of *Nigelia martiale* (≡ *C. martialis*).

#### Cordyceps militaris

Taxon classificationFungiHypocrealesCordycipitaceae

(L.) Fr.

E1B72600-512D-51D0-BCB9-C8A4C2DD7C87

≡ Clavaria militaris L.
≡ Sphaeria militaris (L.) J.F. Gmel.
≡ Hypoxylon militare (L.) Mérat
≡ Xylaria militaris (L.) Gray
≡ Corynesphaera militaris (L.) Dumort.
≡ Torrubia militaris (L.) Tul. & C. Tul.
= Clavaria granulosa Bull.
= Sphaeria militaris var. sphaerocephala J.C. Schmidt
= Cordyceps militaris f. sphaerocephala (J.C. Schmidt) Sacc.
= Cordyceps militaris f. alba Kobayasi & Shimizu ex Y.J. Yao [as ‘albina’]

##### Hosts.

Commonly on Lepidoptera larvae and pupae, infrequently on Hymenoptera (Kobayasi 1941; Kryukov et al. 2011).

##### Distribution.

Widely distributed.

##### Note.

Under laboratory conditions and injection of hyphal bodies into the haemocoel of insects, *C. militaris* can infect many insect orders (Shrestha et al. 2012), including pupae of *Tenebrio molitor* (Tenebrionidae) (De Bary 1867; Sato and Shimazu 2002). Therefore, the conclusion that wireworms (e.g. *Tenebrio molitor*) are the natural hosts of *C. militaris* is probably untenable and we temporarily reject it.

#### Cordyceps nanatakiensis

Taxon classificationFungiHypocrealesCordycipitaceae

Kobayasi & Shimizu

03BD1D7F-FEB4-5175-A4D0-48B7AB8F1AEC

##### Host.

Tenebrionidae larva (Shimizu 1997).

##### Known distribution.

Japan (Kobayasi and Shimizu 1983).

##### Notes.

Taxonomically uncertain species from the previous *Cordyceps*. Its host was originally recorded as a Coleoptera larva (Kobayasi and Shimizu 1983) and then Shimizu (1997) identified it as a Tenebrionidae larva.

#### Cordyceps nirtolii

Taxon classificationFungiHypocrealesCordycipitaceae

Negi, Koranga, Ranj. Singh & Z. Ahmed

63498EA6-1125-53EF-B378-9E1866BBF68A

##### Host.

Larva of Elateridae (*Melanotus communis* (Gyllenhal)).

##### Known distribution.

India (Himalaya) (Negi et al. 2012).

##### Note.

Host of the species was recorded as a larva of *Melanotus communis* (Negi et al. 2012). *Melanotus communis* (Gyllenhal) represents an Elateridae insect, while *Melanotus communis* E. Horak is a mushroom (Agaricales: Strophariaceae).

#### Cordyceps roseostromata

Taxon classificationFungiHypocrealesCordycipitaceae

Kobayasi & Shimizu

02288FB1-36B1-56D7-987F-8B34315848E4

##### Host.

Tenebrionidae larva (Shimizu 1997).

##### Known distribution.

Japan (Kobayasi and Shimizu 1983).

##### Note.

Host of the species was originally recorded as a Coleoptera larva (Kobayasi and Shimizu 1983) and then Shimizu (1997) identified it as a Tenebrionidae larva.

#### Cordyceps rubiginosistipitata

Taxon classificationFungiHypocrealesCordycipitaceae

Kobayasi & Shimizu [as ‘rubiginosostipitata’]

B6A30B17-5A83-5502-BD13-FAB54450C5F3

##### Host.

Tenebrionoidea or Elateroidea larva.

##### Known distribution.

Japan (Kobayasi and Shimizu 1983).

##### Note.

Taxonomically uncertain species from the previous *Cordyceps*. Its host was recorded as a Coleoptera larva (Kobayasi and Shimizu 1983; Shimizu 1997). According to the illustration by Shimizu (1997), the host is a wireworm.

#### Cordyceps rubra

Taxon classificationFungiHypocrealesCordycipitaceae

Möller

B32A8FEC-A6FA-5BEE-8CF8-CA6CA8854370

##### Host.

Elateridae larva (Möller 1901).

##### Known distribution.

Brazil (Möller 1901).

##### Note.

Taxonomically uncertain species from the previous *Cordyceps*.

#### Cordyceps shanxiensis

Taxon classificationFungiHypocrealesCordycipitaceae

B. Liu, Rong & H.S. Jin

F2E24CD6-C89B-5983-8356-7CD4EA4833E5

##### Hosts.

Elateridae larvae (*Melanotus caudex*? and *Pleonomus canaliculatus*?) (Liu et al. 1985).

##### Known distribution.

China (Shanxi) (Liu et al. 1985).

##### Notes.

Taxonomically uncertain species from the previous *Cordyceps*. According to the original description, the species is morphologically similar to *Paraisaria gracilis* (Grev.) Luangsa-ard et al. on Lepidoptera larvae. Notably, the two host names provided by Liu et al. (1985) cannot be retrieved in GBIF (2021).

#### Cordyceps submilitaris

Taxon classificationFungiHypocrealesCordycipitaceae

Henn.

0927BEF2-F114-531B-9862-1C6B501CDDC9

##### Hosts.

Elateroidea or Tenebrionoidea larvae.

##### Known distribution.

South America (Petch 1933).

##### Notes.

Taxonomically uncertain species from the previous *Cordyceps*. Hosts of the species were recorded as beetle larvae in rotten wood (Petch 1933). Petch (1933) considered the species as a synonym of *Nigelia martiale* (≡ *C. martialis*). According to the information given by Petch (1933), hosts of the species are wireworms.

#### Cordyceps velutipes

Taxon classificationFungiHypocrealesCordycipitaceae

Massee

95B8C473-9930-5375-9B73-9EC962BEE314

##### Hosts.

Larvae of Elateridae and Scarabaeidae (*Melolontha* sp.) (Massee 1895; Moureau 1949).

##### Known distribution.

Africa (Massee 1895).

##### Note.

Taxonomically uncertain species from the previous *Cordyceps*.

### Family Clavicipitaceae (Lindau) Earle ex Rogerson, emend. G.H. Sung, J.M. Sung, Hywel-Jones & Spatafora

#### Metarhizium anisopliae

Taxon classificationFungiHypocrealesClavicipitaceae

species complex

C21465F9-47DB-5D8B-8587-3AF41633DC4F

##### Hosts.

More than seven insect orders, including Coleoptera (e.g. Elateridae and Tenebrionidae spp., Kabaluk et al. 2005, 2017; Humber and Hansen 2005; Reddy et al. 2014); inhabiting soil, plant surfaces and plant internal tissues (Hu et al. 2014; Bamisile et al. 2018; Brunner-Mendoza et al. 2019).

##### Distribution.

Widely distributed.

##### Note.

*Metarhizium anisopliae* species complex includes several cryptic species, for example, *M. anisopliae* (Metschn.) Sorokīn, *M. brunneum* Petch and *M. robertsii* J.F. Bisch., S.A. Rehner & Humber (Bischoff et al. 2009; Kepler et al. 2014; Mongkolsamrit et al. 2020). Amongst them, *M. brunneum* was most often noted as a wireworm pathogen (e.g. Kabaluk et al. 2017).

#### Metarhizium atrovirens

Taxon classificationFungiHypocrealesClavicipitaceae

(Kobayasi & Shimizu) Kepler, S.A. Rehner & Humber

25C6B798-656B-5888-82C2-1D95C5980F1F

≡ Cordyceps atrovirens Kobayasi & Shimizu
≡ Metacordyceps atrovirens (Kobayasi & Shimizu) Kepler, G.H. Sung & Spatafora

##### Hosts.

Tenebrionidae larvae (Shimizu 1997).

##### Known distribution.

Japan (Kobayasi and Shimizu 1978; Shimizu 1997).

##### Note.

Hosts of the species were originally recorded as Coleoptera larvae (Kobayasi and Shimizu 1978) and then Shimizu (1997) identified them as Tenebrionidae larvae.

#### Metarhizium brachyspermum

Taxon classificationFungiHypocrealesClavicipitaceae

Koh. Yamam., Ohmae & Orihara

C68F3147-8A85-54F5-A673-5E2513B963A7

##### Hosts.

Elateridae larvae and pupae (Yamamoto et al. 2020).

##### Known distribution.

Japan (Yamamoto et al. 2020).

#### Metarhizium campsosterni

Taxon classificationFungiHypocrealesClavicipitaceae

(W.M. Zhang & T.H. Li) Kepler, S.A. Rehner & Humber

6FC926DE-38C7-51B8-8906-9E7FF29C712B

≡ Cordyceps campsosterni W.M. Zhang & T.H. Li [as ‘campsosterna’]
≡ Metacordyceps campsosterni (W.M. Zhang & T.H. Li) G.H. Sung, J.M. Sung, Hywel-Jones & Spatafora

##### Hosts.

Larva and adult of *Campsosternus auratus* (Elateridae) (Zhang et al. 2004).

##### Known distribution.

China (Guangdong) (Zhang et al. 2004).

#### Metarhizium clavatum

Taxon classificationFungiHypocrealesClavicipitaceae

Luangsa-ard, Mongkolsamrit, Lamlertthon, Thanakitpipattana & Samson

39EFEAAF-D792-576D-94EA-A3CD6B97AB78

##### Hosts.

Elateridae (*Oxynopterus*) larvae (Mongkolsamrit et al. 2020).

##### Known distribution.

Thailand (Mongkolsamrit et al. 2020).

#### Metarhizium flavum

Taxon classificationFungiHypocrealesClavicipitaceae

Luangsa-ard, Mongkolsamrit, Thanakitpipattana & Samson

726B8AEF-D11A-590E-8D1A-CB71B21385C6

##### Hosts.

Tenebrionoidea or Elateroidea larvae.

##### Known distribution.

Thailand (Mongkolsamrit et al. 2020).

##### Note.

Hosts of the species were originally recorded as Coleoptera larvae (Mongkolsamrit et al. 2020). According to the illustration and the information provided, the hosts are wireworms.

#### Metarhizium kalasinense

Taxon classificationFungiHypocrealesClavicipitaceae

Tasan., Khons., Thanakitp., Mongkols. & Luangsa-ard

1D197475-31B9-522B-9E8F-6D2150A24B23

##### Hosts.

Elateroidea larvae.

##### Known distribution.

Thailand (Luangsa-ard et al. 2017).

##### Note.

Hosts of the species were originally recorded as elaterid larvae (Coleoptera) (Luangsa-ard et al. 2017).

#### Metarhizium pseudoatrovirens

Taxon classificationFungiHypocrealesClavicipitaceae

(Kobayasi & Shimizu) Kepler, S.A. Rehner & Humber

DA448079-E062-512B-8744-687D0739271A

≡ Cordyceps pseudoatrovirens Kobayasi & Shimizu
≡ Metacordyceps pseudoatrovirens (Kobayasi & Shimizu) Kepler, G.H. Sung & Spatafora

##### Hosts.

Larvae of Tenebrionoidea and/or Elateroidea (Shimizu 1997; Liang 2007).

##### Known distribution.

China (Guizhou), Japan (Kobayasi and Shimizu 1982b; Liang 2007).

##### Notes.

The host of the species was originally recorded as a Coleoptera larva (Kobayasi and Shimizu 1982b), then Shimizu (1997) identified it as a Tenebrionidae larva. Liang (2007) recorded the species with pictures (four specimens) and wireworm hosts.

#### Metarhizium purpureonigrum

Taxon classificationFungiHypocrealesClavicipitaceae

Luangsa-ard, Tasanathai, Thanakitpipattana & Samson

14BE0100-9809-5E20-A213-32472B76CA52

##### Hosts.

Elateridae larvae (*Campsosternus* sp.).

##### Known distribution.

Thailand (Mongkolsamrit et al. 2020).

##### Notes.

According to the description and pictures provided (Mongkolsamrit et al. 2020), the species is probably a synonym of *O. jiangxiensis*, a traditional Chinese medicinal mushroom (Zha et al. 2018, also see *O. jiangxiensis* below). Hosts of the species, which were recorded as Coleoptera larvae, are Elateridae larvae (*Campsosternus* sp.).

#### Metarhizium purpureum

Taxon classificationFungiHypocrealesClavicipitaceae

Luangsa-ard, Mongkolsamrit, Lamlertthon Thanakitpipattana & Samson

90C1F52B-4146-5034-8C3E-68C01F6F07B8

##### Hosts.

Elateridae (*Oxynopterus*) larvae (Mongkolsamrit et al. 2020).

##### Known distribution.

Thailand (Mongkolsamrit et al. 2020).

#### Nigelia martiale

Taxon classificationFungiHypocrealesClavicipitaceae

(Speg.) Luangsa-ard & Thanakitp.

BF3530DB-2888-5AEA-B35D-A7AD1D74E8C7

≡ Cordyceps martialis Speg.
≡ Metacordyceps martialis (Speg.) Kepler, G.H. Sung & Spatafora
≡ Metarhizium martiale (Speg.) Kepler, S.A. Rehner & Humber

##### Hosts.

Larvae of Coleoptera (e.g. Elateridae, Shrestha et al. 2016; Cerambycidae, Spegazzini 1889) and Lepidoptera (Liang 2007; Kepler et al. 2012).

##### Known distribution.

Brazil, China (Guangdong, Zhejiang, Taiwan), the West Indies (Kobayasi 1941; Liang 2007).

### Family Ophiocordycipitaceae G.H. Sung, J.M. Sung, Hywel-Jones & Spatafora

#### Ophiocordyceps acicularis

Taxon classificationFungiHypocrealesOphiocordycipitaceae

(Ravenel) Petch

AA83A544-05E3-544A-ACBB-2A17A275435F

≡ Cordyceps acicularis Ravenel

##### Hosts.

Elateridae larvae (Shimizu 1997).

##### Known distribution.

China (Jiangsu, Guangdong, Guizhou, Hainan, Taiwan), Japan, Russia (Far East), U.S.A. (Carolina) (Massee 1895; Kobayasi and Shimizu 1980a, Koval 1984; Liang 2007).

##### Note.

Hosts of the species were generally identified as wireworms or Coleoptera larvae (Kobayasi and Shimizu 1980a, Liang 2007). Shimizu (1997) identified the hosts of the species from Japan and Taiwan as Elateridae larvae.

#### Ophiocordyceps agriotis

Taxon classificationFungiHypocrealesOphiocordycipitaceae

(Kawam.) G.H. Sung, J.M. Sung, Hywel-Jones & Spatafora [as ‘agriotidis’]

E6DDCE30-F732-587A-B7C6-7FFAC7907E34

≡ Cordyceps agriota Kawam. [as ‘agriotidis’ in Index Fungorum (2021)]

##### Hosts.

Elateridae (e.g. *Agriotes*) larvae (Kobayasi and Shimizu 1980a, Shimizu 1997).

##### Known distribution.

China (Guizhou, Jilin), Japan (Kobayasi and Shimizu 1980a, Yang 2004; Liang 2007).

##### Notes.

The specific epithet of this species was adopted from the generic name of its host insect ‘*Agriotes*’ (Kobayasi and Shimizu 1980a). The epithet ‘agriotidis’, used in Index Fungorum (2021) and related literature (e.g. Sung et al. 2007), is incorrect. Yang (2004) and Liang (2007) also recorded its hosts as Elateridae larvae.

#### Ophiocordyceps annulata

Taxon classificationFungiHypocrealesOphiocordycipitaceae

(Kobayasi & Shimizu) Spatafora, Kepler & C.A. Quandt [as ‘annulata’ in Index Fungorum (2021)]

3CA94FCE-3600-5F96-9C1B-46B10C145AE4

≡ Cordyceps annulata Kobayasi & Shimizu [as ‘annulata’ in Index Fungorum (2021)]

##### Host.

Tenebrionoidea or Elateroidea larva.

##### Known distribution.

Japan (Kobayasi and Shimizu 1982a).

##### Note.

Host of the species was originally recorded as a Coleoptera larva (Kobayasi and Shimizu 1982a). According to the illustration by Shimizu (1997), the host is a wireworm.

#### Ophiocordyceps appendiculata

Taxon classificationFungiHypocrealesOphiocordycipitaceae

(Kobayasi & Shimizu) G.H. Sung, J.M. Sung, Hywel-Jones & Spatafora

10B970D3-CDDA-555E-A488-C8D9459CF229

≡ Cordyceps appendiculata Kobayasi & Shimizu

##### Host.

Tenebrionidae larva (Shimizu 1997).

##### Known distribution.

Japan (Kobayasi and Shimizu 1983).

##### Note.

Host of the species was originally recorded as a Coleoptera larva (Kobayasi and Shimizu 1983). Shimizu (1997) identified it as a Tenebrionidae larva.

#### Ophiocordyceps asyuensis

Taxon classificationFungiHypocrealesOphiocordycipitaceae

(Kobayasi & Shimizu) G.H. Sung, J.M. Sung, Hywel-Jones & Spatafora [as ‘asyuënsis’]

46AFFF81-4FD5-56A8-A1BF-9E0066707D3C

≡ Cordyceps asyuensis Kobayasi & Shimizu

##### Hosts.

Elateroidea or Tenebrionoidea larva.

##### Known distribution.

Japan (Kobayasi and Shimizu 1980b).

##### Note.

Host of the species was originally recorded as a Coleoptera larva (Kobayasi and Shimizu 1980b). According to the illustration by Shimizu (1997), the host is a wireworm.

#### Ophiocordyceps brunneipunctata

Taxon classificationFungiHypocrealesOphiocordycipitaceae

(Hywel-Jones) G.H. Sung, J.M. Sung, Hywel-Jones & Spatafora

23663851-7A75-530E-A45B-DEEE633C6D0B

≡ Cordyceps brunneipunctata Hywel-Jones [as ‘brunneapunctata’]

##### Hosts.

Elateridae larvae (Hywel-Jones 1995).

##### Known distribution.

Thailand (Hywel-Jones 1995).

#### Ophiocordyceps clavata

Taxon classificationFungiHypocrealesOphiocordycipitaceae

(Kobayasi & Shimizu) G.H. Sung, J.M. Sung, Hywel-Jones & Spatafora

F2CB7E54-C9AB-5081-A5C8-41900D3A4A2E

≡ Cordyceps clavata Kobayasi & Shimizu

##### Hosts.

Tenebrionidae larvae (Shimizu 1997).

##### Known distribution.

Japan (Shimizu 1997).

##### Note.

The host of the species was originally recorded as a Coleoptera larva (Kobayasi and Shimizu 1980b). Shimizu (1997) identified the hosts of the species as Tenebrionidae larvae.

#### Ophiocordyceps elateridicola

Taxon classificationFungiHypocrealesOphiocordycipitaceae

(Kobayasi & Shimizu) G.H. Sung, J.M. Sung, Hywel-Jones & Spatafora

65430831-127E-576F-BEA0-B9A1B2F4FD74

≡ Cordyceps elateridicola Kobayasi & Shimizu

##### Host.

Elateridae larvae (Kobayasi and Shimizu 1983; Shimizu 1997).

##### Known distribution.

China (Taiwan), Japan (Shimizu 1997).

#### Ophiocordyceps entomorrhiza

Taxon classificationFungiHypocrealesOphiocordycipitaceae

(Dicks.) G.H. Sung, J.M. Sung, Hywel-Jones & Spatafora

BEAD4EA1-35CE-50DC-8834-FE4E19CC5FD4

≡ Sphaeria entomorrhiza Dicks.
≡ Xylaria entomorrhiza (Dicks.) Gray
≡ Cordyceps entomorrhiza (Dicks.) Fr.
= Isaria eleutheratorum Nees
= Torrubia cinerea Tul. & C. Tul.
= Cordyceps cinerea (Tul. & C. Tul.) Sacc.
= Cordyceps meneristitis F. Muell. & Berk. [as ‘menesteridis’]
= Cordyceps entomorrhiza var. meneristitis (F. Muell. & Berk.) Cooke [as ‘mesenteridis’]
= Cordyceps carabi Quél.
= Tilachlidiopsis nigra Yakush. & Kumaz.
= Hirsutella eleutheratorum (Nees) Petch

##### Hosts.

Larvae and adults of many Coleoptera families, for example, Tenebrionidae larva (Shrestha et al. 2016) and Lampyridae larvae.

##### Distribution.

Widely distributed.

##### Note.

According to the illustrations by Shimizu (1997), we identify the hosts of the species from Japan as Lampyridae larvae (Elateroidea).

#### Ophiocordyceps falcatoides

Taxon classificationFungiHypocrealesOphiocordycipitaceae

(Kobayasi & Shimizu) G.H. Sung, J.M. Sung, Hywel-Jones & Spatafora

C1F6D2A3-74B2-5DC7-8B77-3C9CDA7B2601

≡ Cordyceps falcatoides Kobayasi & Shimizu

##### Host.

Tenebrionoidea or Elateroidea larva.

##### Known distribution.

Japan (Kobayasi and Shimizu 1980a).

##### Note.

Host of the species was originally recorded as a Coleoptera larva (Kobayasi and Shimizu 1980a). According to the illustration by Shimizu (1997), the host is a wireworm.

#### Ophiocordyceps ferruginosa

Taxon classificationFungiHypocrealesOphiocordycipitaceae

(Kobayasi & Shimizu) G.H. Sung, J.M. Sung, Hywel-Jones & Spatafora

E1F98161-D3D6-55C9-AE39-97C66A7D5A9A

≡ Cordyceps ferruginosa Kobayasi & Shimizu

##### Hosts.

Xylophagidae larvae (Diptera).

##### Known distribution.

Japan (Kobayasi and Shimizu 1980b).

##### Notes.

Hosts of the species were originally identified as Coleoptera larvae living in decayed wood (Kobayasi and Shimizu 1980b, Shimizu 1997). According to the illustrations by Shimizu (1997), the hosts are actually Diptera (Xylophagidae) larvae. Considering the very similar morphology and the same hosts between *O. ferruginosa* and *O. variabilis*, the former might be a synonym of the latter (see notes of *O. variabilis* below). As a result, *O. ferruginosa* is not a pathogen of wireworms.

#### Ophiocordyceps formosana

Taxon classificationFungiHypocrealesOphiocordycipitaceae

(Kobayasi & Shimizu) Yen W. Wang, S.H. Tsai, Tzean & T.L. Shen

41EEC6C2-91FE-5441-B844-A7516D2A361F

≡ Cordyceps formosana Kobayasi & Shimizu

##### Hosts.

Tenebrionoidea larvae (Li et al. 2002, 2016).

##### Known distribution.

China (Anhui, Fujian, Hunan, Taiwan) (Kobayasi and Shimizu 1981; Li et al. 2002, 2016).

##### Notes.

The host of the species was originally recorded as a Coleoptera larva (Kobayasi and Shimizu 1981). According to the illustration by Shimizu (1997), it appears to be a Tenebrionoidea larva. Li et al. (2002) identified the host of their collection as a Tenebrionidae larva. We cautiously identify these hosts as Tenebrionoidea larvae (used in Li et al. 2016).

#### Ophiocordyceps jiangxiensis

Taxon classificationFungiHypocrealesOphiocordycipitaceae

(Z.Q. Liang, A.Y. Liu & Yong C. Jiang) G.H. Sung, J.M. Sung, Hywel-Jones & Spatafora

4CCCC473-245D-50FB-A92E-7F59B6F9926B

≡ Cordyceps jiangxiensis Z.Q. Liang, A.Y. Liu & Yong C. Jiang

##### Hosts.

Elateridae larvae (*Campsosternus* sp.) (Liang et al. 2001; Zha et al. 2018).

##### Known distribution.

China (Jiangxi, Fujian, Yunnan) (Zha et al. 2018).

##### Notes.

The species was originally described by Liang et al. (2001) with specimens from Jiangxi, China. Sung et al. (2007) revised it to *O. jiangxiensis* only based on the original morphological description. The species is closely similar to *Metarhizium purpureonigrum*, a recently-described species from Thailand (Mongkolsamrit et al. 2020). Future studies are warranted to clarify its taxonomic placement.

#### Ophiocordyceps larvicola

Taxon classificationFungiHypocrealesOphiocordycipitaceae

(Quél.) Van Vooren

C6C9A018-9C25-5C2E-BAE2-D6B15E5CABB4

≡ Cordyceps larvicola Quél.

##### Hosts.

Larvae of Cerambycidae, Scarabaeidae and Tenebrionidae (e.g. *Cylindronotus* sp., *Helops* spp.) (Kobayasi 1941; Shrestha et al. 2016).

##### Known distribution.

France (Kobayasi 1941), the European part of Russia (Koval 1984).

#### Ophiocordyceps melolonthae

Taxon classificationFungiHypocrealesOphiocordycipitaceae

(Tul. & C. Tul.) G.H. Sung, J.M. Sung, Hywel-Jones & Spatafora

8443567F-3B33-536A-9D97-E0C51EB6490D

≡ Torrubia melolonthae Tul. & C. Tul.
≡ Cordyceps melolonthae (Tul. & C. Tul.) Sacc.
= Cordyceps rickii Lloyd
= Cordyceps melolonthae var. rickii (Lloyd) Mains
= Ophiocordyceps melolonthae var. rickii (Lloyd) G.H. Sung, J.M. Sung, Hywel-Jones & Spatafora

##### Hosts.

Scarabaeidae larvae (Shrestha et al. 2016), Elateridae larvae (Shimizu 1997).

##### Distribution.

North, Central and South America, the West Indies (Kobayasi 1941; Mains 1958), Japan (Shimizu 1997), Belarus, the Russian Far East (Koval 1984).

#### Ophiocordyceps nigripoda

Taxon classificationFungiHypocrealesOphiocordycipitaceae

(Kobayasi & Shimizu) G.H. Sung, J.M. Sung, Hywel-Jones & Spatafora [as ‘nigripes’]

A4FE6097-7352-527D-83A0-CF92CD67560F

≡ Cordyceps nigripoda Kobayasi & Shimizu

##### Host.

Elateroidea or Tenebrionoidea larva.

##### Known distribution.

Japan (Kobayasi and Shimizu 1982b).

##### Note.

Host of the species was originally recorded as a Coleoptera larva (Kobayasi and Shimizu 1982b). According to the illustration by Shimizu (1997), the host is a wireworm.

#### Ophiocordyceps purpureostromata

Taxon classificationFungiHypocrealesOphiocordycipitaceae

(Kobayasi) G.H. Sung, J.M. Sung, Hywel-Jones & Spatafora

47BDC6A0-7023-5151-8FED-63B3C86C43F5

≡ Cordyceps purpureostromata Kobayasi
= Cordyceps purpureostromata f. recurvata Kobayasi
= Ophiocordyceps purpureostromata f. recurvata (Kobayasi) G.H. Sung, J.M. Sung, Hywel-Jones & Spatafora

##### Hosts.

Elateridae larvae (Shimizu 1997).

##### Known distribution.

Japan (Kobayasi and Shimizu 1980b).

#### Ophiocordyceps rubiginosiperitheciata

Taxon classificationFungiHypocrealesOphiocordycipitaceae

(Kobayasi & Shimizu) G.H. Sung, J.M. Sung, Hywel-Jones & Spatafora

70807050-4735-575A-942E-42D4E9137A64

≡ Cordyceps rubiginosiperitheciata Kobayasi & Shimizu [as ‘rubiginosoperitheciata’]

##### Hosts.

Elateroidea or Tenebrionoidea larvae.

##### Known distribution.

Japan (Shimizu 1997).

##### Note.

The host of the species was originally recorded as a Coleoptera larva (Kobayasi and Shimizu 1983). According to the illustration by Shimizu (1997), hosts of the species are wireworms.

#### Ophiocordyceps rubripunctata

Taxon classificationFungiHypocrealesOphiocordycipitaceae

(Moreau) G.H. Sung, J.M. Sung, Hywel-Jones & Spatafora

820C108C-C5FF-54BA-95A5-27837268E80C

≡ Cordyceps rubripunctata Moreau
= Hirsutella rubripunctata Samson, H.C. Evans & Hoekstra

##### Hosts.

Elateridae larvae (Samson et al. 1982).

##### Known distribution.

Congo, Ghana (Samson et al. 1982).

#### Ophiocordyceps salebrosa

Taxon classificationFungiHypocrealesOphiocordycipitaceae

(Mains) G.H. Sung, J.M. Sung, Hywel-Jones & Spatafora

CD1FD542-C2A9-54B9-ADC6-B75C0338C0AE

≡ Cordyceps salebrosa Mains

##### Host.

Elateridae adult (Mains 1947).

##### Known distribution.

Panama Canal Zone (Barro Colorado Island) (Mains 1947).

##### Note.

Notably, the host of the species is an adult.

#### Ophiocordyceps sporangifera

Taxon classificationFungiHypocrealesOphiocordycipitaceae

Y.P. Xiao, T.C. Wen & K.D. Hyde

A3E42605-DE06-5B7A-9431-2FD02B7609F1

##### Host.

Elateroidea or Tenebrionoidea larva.

##### Known distribution.

Thailand (Xiao et al. 2019).

##### Note.

The host of the species was originally identified as an Elateridae larva (Xiao et al. 2019).

#### Ophiocordyceps stylophora

Taxon classificationFungiHypocrealesOphiocordycipitaceae

(Berk. & Broome) G.H. Sung, J.M. Sung, Hywel-Jones & Spatafora

B4CEFD1C-6B66-5125-B9B3-40DEB4A498C3

≡ Cordyceps stylophora Berk. & Broome
= Hirsutella stylophora Mains

##### Hosts.

Larvae of Coleoptera (Cerambycidae, Elateridae, Scarabaeidae) (Shrestha et al. 2016).

##### Known distribution.

Canada (Nova Scotia), China (Guangxi, Jilin, Zhejiang), Japan, Russia (Far East), U.S.A. (Carolina) (Kobayasi 1941; Mains 1941; Koval 1984; Liang 2007).

##### Note.

Liang (2007) recorded the hosts of the species as Lepidoptera larvae, but his provided picture (a specimen collected from Jilin, China) appears to be a wireworm host.

#### Ophiocordyceps subflavida

Taxon classificationFungiHypocrealesOphiocordycipitaceae

(Mains) G.H. Sung, J.M. Sung, Hywel-Jones & Spatafora

BBEFC256-F6FD-57F8-8FD0-E7CEAA51B9E1

≡ Cordyceps albida Pat. & Gaillard
≡ Cordyceps subflavida Mains

##### Hosts.

Elateridae larvae (Shimizu 1997).

##### Known distribution.

Japan (Shimizu 1997), Venezuela (Mains 1959).

##### Note.

The species was originally reported from Venezuela and its host was recorded as an insect larva (Mains 1959). Shimizu (1997) identified the host of a specimen from Japan as an Elateridae larva.

#### Ophiocordyceps variabilis

Taxon classificationFungiHypocrealesOphiocordycipitaceae

(Petch) G.H. Sung, J.M. Sung, Hywel-Jones & Spatafora

16FD06D6-47E1-5F3E-8E2B-D20E5A7E3BBC

≡ Cordyceps variabilis Petch
= Cordyceps viperina Mains

##### Hosts.

Xylophagidae larvae (Diptera) (Hodge et al. 1998; Yaroslavtseva et al. 2019).

##### Known distribution.

China (Shaanxi),Europe, Russia (Far East, Western Siberia), North America (Petch 1937; Liang 2007; Hodge et al. 1998; Yaroslavtseva et al. 2019).

##### Notes.

In early literature, *O. variabilis* was recorded on Coleoptera (e.g. Elateridae) and Diptera larvae in rotten wood (Petch 1937; Mains 1958; Liang 2007). Hodge et al. (1998) checked many samples and confirmed the hosts to be Xylophagidae larvae (Diptera). More than 40 samples of *O. variabilis* were collected in Russia (Far East, Western Siberia) and all of them developed on Xylophagidae larvae (Yaroslavtseva et al. 2019; Kryukov et al., unpublished). Ecological habits and morphology of Xylophagidae larvae and wireworms are closely similar, but their last abdominal segments are distinctly different. As with *O. ferruginosa* listed above, we conclude that *O. variabilis* is not a pathogen of wireworms.

#### Paraisaria gracilioides

Taxon classificationFungiHypocrealesOphiocordycipitaceae

(Kobayasi) C.R. Li, M.Z. Fan & Z.Z. Li

D37F4C78-7771-515B-8555-852150AFD17E

≡ Isaria gracilioides Kobayasi
= Cordyceps gracilioides Kobayasi
= Ophiocordyceps gracilioides (Kobayasi) G.H. Sung, J.M. Sung, Hywel-Jones & Spatafora
= Paraisaria gracilioides (Kobayasi) Luangsa-ard, Mongkolsamrit & Samson, **syn. nov.**

##### Hosts.

Elateridae larvae (Shimizu 1997; Yahagi 2008).

##### Known distribution.

China (Anhui, Fujian), Japan, Russia (Far East) (Kobayasi 1941; Koval 1984; Liang 2007).

##### Notes.

The species is similar to *Paraisaria gracilis* (Grev.) Luangsa-ard et al., but the former grows on Coleoptera larvae (Elateridae), while the latter on Lepidoptera larvae (Kobayasi 1941; Yahagi 2008). Hosts of the sexual *C. gracilioides* and its asexual *Isaria gracilioides* were both originally mistakenly identified as Cossidae larvae (Lepidoptera instead of Coleoptera) (Kobayasi 1941). Fan et al. (2001) collected a sexual specimen of the species on a Coleoptera larva (wireworm); Li et al. (2004) successfully isolated its asexual morph and revised the asexual *Isaria gracilioides* to the asexual *Paraisaria gracilioides* (Kobayasi) C.R. Li et al., linked with the sexual *C. gracilioides*. Later, the sexual *C. gracilioides* has been revised in an orderly manner to *O. gracilioides* (Sung et al. 2007) and *Paraisaria gracilioides* (Kobayasi) Luangsa-ard et al. (Mongkolsamrit et al. 2019). Considering the rules of priority and one fungus, one name (Kepler et al. 2013), we combine *Paraisaria gracilioides* (Kobayasi) Luangsa-ard et al. with *Paraisaria gracilioides* (Kobayasi) C.R. Li et al.

#### Paraisaria phuwiangensis

Taxon classificationFungiHypocrealesOphiocordycipitaceae

Mongkolsamrit, Noisripoom, Himaman, Jangsantear & Luangsa-ard

D5771BFE-EB02-5CD4-B586-3499900ECC0D

##### Hosts.

Elateridae larvae (Mongkolsamrit et al. 2019).

##### Known distribution.

Thailand (Mongkolsamrit et al. 2019).

#### Paraisaria yodhathaii

Taxon classificationFungiHypocrealesOphiocordycipitaceae

Mongkolsamrit, Noisripoom, Lamlertthon & Luangsa-ard

28B0E69E-18BC-598C-889B-95CDDC13BD0F

##### Hosts.

Elateridae larva (Mongkolsamrit et al. 2019).

##### Known distribution.

Thailand (Mongkolsamrit et al. 2019).

#### Perennicordyceps cuboidea

Taxon classificationFungiHypocrealesOphiocordycipitaceae

(Kobayasi & Shimizu) Matočec & I. Kušan

75CB1B77-B58E-575F-8963-62D205DBC7C8

≡ Cordyceps cuboidea Kobayasi & Shimizu
≡ Ophiocordyceps cuboidea (Kobayasi & Shimizu) S. Ban, Sakane & Nakagiri
≡ Polycephalomyces cuboideus (Kobayasi & Shimizu) Kepler & Spatafora
= Cordyceps alboperitheciata Kobayasi & Shimizu

##### Hosts.

Tenebrionoidea and/or Elateroidea larvae (Shimizu 1997; Ban et al. 2009); stroma of *O. stylophora* (Ban et al. 2009).

##### Known distribution.

Japan (Kobayasi and Shimizu 1980b).

##### Note.

The host of the species was originally recorded as a Coleoptera larva (Kobayasi and Shimizu 1980b). According to the illustrations by Shimizu (1997) and Ban et al. (2009), hosts of the species are wireworms.

#### Perennicordyceps ryogamiensis

Taxon classificationFungiHypocrealesOphiocordycipitaceae

(Kobayasi & Shimizu) Matočec & I. Kušan

BF250D66-243D-545E-AAA2-9EF058D685B3

≡ Cordyceps ryogamiensis Kobayasi & Shimizu
≡ Ophiocordyceps ryogamiensis (Kobayasi & Shimizu) G.H. Sung, J.M. Sung, Hywel-Jones & Spatafora
≡ Polycephalomyces ryogamiensis (Kobayasi & Shimizu) Kepler & Spatafora

##### Host.

Tenebrionoidea larva.

##### Known distribution.

Japan (Kobayasi and Shimizu 1983).

##### Note.

Host of the species was originally recorded as a Coleoptera larva (Kobayasi and Shimizu 1983). According to the illustration by Shimizu (1997), the host is a Tenebrionoidea larva.

#### Polycephalomyces phaothaiensis

Taxon classificationFungiHypocrealesOphiocordycipitaceae

Mongkols., Noisrip., Lamlertthon & Luangsa-ard

BBA5D6B0-2F5B-5BE0-8DA7-95219EEF19C9

##### Hosts.

Tenebrionoidea or Elateroidea larvae.

##### Known distribution.

Thailand (Crous et al. 2017).

##### Note.

Hosts of the species were recorded as Coleoptera larvae (Crous et al. 2017). According to the picture provided, the hosts are wireworms.

#### Tolypocladium cylindrosporum

Taxon classificationFungiHypocrealesOphiocordycipitaceae

W. Gams

C66B32A3-D0D3-5018-8A3F-7E6C464363A5

≡ Beauveria cylindrospora (W. Gams) Arx

##### Hosts.

Coleoptera (e.g. Elateridae sp.), Diptera, Hymenoptera and Lepidoptera (Humber and Hansen 2005); inhabit soil (Scorsetti et al. 2012).

##### Distribution.

Widely distributed.

#### Tolypocladium inflatum

Taxon classificationFungiHypocrealesOphiocordycipitaceae

W. Gams

83B3D922-C113-5B73-A88D-31729B2B431E

= Pachybasium niveum O. Rostr.
= Tolypocladium niveum (O. Rostr.) Bissett
= Cordyceps subsessilis Petch
= Elaphocordyceps subsessilis (Petch) G.H. Sung, J.M. Sung & Spatafora
= Cordyceps facis Kobayasi & Shimizu [as ‘Codyceps’]

##### Hosts.

Tenebrionidae larvae (Shimizu 1997).

##### Distribution.

Widely distributed (Petch 1937; Kobayasi 1982; Sung et al. 2007).

##### Note.

Hosts of the species were previously recorded as Coleoptera larvae (Petch 1937; Kobayasi 1982). Shimizu (1997) identified them as Tenebrionidae larvae.

## Discussion

The superfamilies Elateroidea and Tenebrionoidea are two very large groups of beetles and comprise more than 50 families of Coleoptera (Catalogue of Life 2021). These include Lampyridae (fireflies), Elateridae (click beetles), Phengodidae (glowworm beetles), Cantharidae (soldier beetles) and their relatives in Elateroidea; and Meloidae (blister beetles), Anthicidae (ant-like flower beetles), Mordellidae (tumbling flower beetles), Tenebrionidae (darkling beetle), Ciidae (the minute tree-fungus beetles), Zopheridae (ironclad beetles) and their relatives in Tenebrionoidea. Most of Elateroidea and Tenebrionoidea larvae (wireworms) are closely similar and morphology alone could hardly distinguish them. In practice, hosts of many wireworm-infecting *Cordyceps* s.l. species are commonly identified as Elateridae (mainly) or Tenebrionidae larvae. Considering the difficulties in identifying wireworms, we suggest to use the superfamily names (Elateroidea or Tenebrionoidea) to record the hosts of the fungi, unless we can definitely know the species identity (e.g. by barcoding techniques).

In present paper, we summarised the data of wireworm-infecting species of *Cordyceps* s.l. To date, a total of 63 species have been reported, including 17 species (*Akanthomyces*, *Beauveria* and *Cordyceps*) in Cordycipitaceae, 11 species (*Metarhizium* and *Nigelia*) in Clavicipitaceae and 35 species (*Ophiocordyceps*, *Paraisaria*, *Perennicordyceps*, *Polycephalomyces* and *Tolypocladium*) in Ophiocordycipitaceae. Amongst these, *C. militaris*, *O. ferruginosa* and *O. variabilis* are rejected; the remaining 60 species are accepted as natural pathogens of wireworms. It is likely that a significant portion of fungi, associated with wireworms, is represented by specialised forms. Thirteen of the reported species (20%) have broad host ranges, that is, they can infect different arthropod taxa and may also parasitise fungi and nematodes. The other 47 species (80%) have, thus far, been registered on wireworms only. Generalist fungi are mostly widespread, whereas specialised fungi are generally reported from warm and humid environments of Southeast Asia (Japan, south-western China and Thailand), the Amazon of South America and the Russian Far East. It should be noted that many animal-associated fungi are awaiting description, especially in groups, such as Hypocreales (Antonelli et al. 2020; Cheek et al. 2020) and many taxonomically-uncertain *Cordyceps* s.l. species infecting Elateroidea and Tenebrionoidea remain to be studied. Apart from the description of novel taxa, further studies should focus on revisions of these uncertain species and further information of wireworm hosts. Limited by lack of information and taxonomic knowledge of larvae, species diversity of wireworm-infecting *Cordyceps* s.l. may not have been completely accounted for and many wireworm hosts cannot be or are incorrectly assigned to their families.

This is the first study summarising species diversity of wireworm-infecting *Cordyceps* s.l. A checklist of 60 species is provided and two novel species are described. Our work provides basic information for future research on species diversity of *Cordyceps* s.l. associated with wireworms, management and biocontrol of wireworm populations, as well as on edible and medicinal insects and fungi.

## Supplementary Material

XML Treatment for Ophiocordyceps borealis

XML Treatment for Ophiocordyceps spicatus

XML Treatment for Polycephalomyces formosus

XML Treatment for Akanthomyces lecanii

XML Treatment for Beauveria bassiana

XML Treatment for Cordyceps aurantiaca

XML Treatment for Cordyceps chiangdaoensis

XML Treatment for Cordyceps chishuiensis

XML Treatment for Cordyceps farinosa

XML Treatment for Cordyceps fumosorosea

XML Treatment for Cordyceps huntii

XML Treatment for Cordyceps militaris

XML Treatment for Cordyceps nanatakiensis

XML Treatment for Cordyceps nirtolii

XML Treatment for Cordyceps roseostromata

XML Treatment for Cordyceps rubiginosistipitata

XML Treatment for Cordyceps rubra

XML Treatment for Cordyceps shanxiensis

XML Treatment for Cordyceps submilitaris

XML Treatment for Cordyceps velutipes

XML Treatment for Metarhizium anisopliae

XML Treatment for Metarhizium atrovirens

XML Treatment for Metarhizium brachyspermum

XML Treatment for Metarhizium campsosterni

XML Treatment for Metarhizium clavatum

XML Treatment for Metarhizium flavum

XML Treatment for Metarhizium kalasinense

XML Treatment for Metarhizium pseudoatrovirens

XML Treatment for Metarhizium purpureonigrum

XML Treatment for Metarhizium purpureum

XML Treatment for Nigelia martiale

XML Treatment for Ophiocordyceps acicularis

XML Treatment for Ophiocordyceps agriotis

XML Treatment for Ophiocordyceps annulata

XML Treatment for Ophiocordyceps appendiculata

XML Treatment for Ophiocordyceps asyuensis

XML Treatment for Ophiocordyceps brunneipunctata

XML Treatment for Ophiocordyceps clavata

XML Treatment for Ophiocordyceps elateridicola

XML Treatment for Ophiocordyceps entomorrhiza

XML Treatment for Ophiocordyceps falcatoides

XML Treatment for Ophiocordyceps ferruginosa

XML Treatment for Ophiocordyceps formosana

XML Treatment for Ophiocordyceps jiangxiensis

XML Treatment for Ophiocordyceps larvicola

XML Treatment for Ophiocordyceps melolonthae

XML Treatment for Ophiocordyceps nigripoda

XML Treatment for Ophiocordyceps purpureostromata

XML Treatment for Ophiocordyceps rubiginosiperitheciata

XML Treatment for Ophiocordyceps rubripunctata

XML Treatment for Ophiocordyceps salebrosa

XML Treatment for Ophiocordyceps sporangifera

XML Treatment for Ophiocordyceps stylophora

XML Treatment for Ophiocordyceps subflavida

XML Treatment for Ophiocordyceps variabilis

XML Treatment for Paraisaria gracilioides

XML Treatment for Paraisaria phuwiangensis

XML Treatment for Paraisaria yodhathaii

XML Treatment for Perennicordyceps cuboidea

XML Treatment for Perennicordyceps ryogamiensis

XML Treatment for Polycephalomyces phaothaiensis

XML Treatment for Tolypocladium cylindrosporum

XML Treatment for Tolypocladium inflatum
